# Knowledge on risk factors and practice of early detection methods of breast cancer among graduating students of Debre Tabor University, Northcentral Ethiopia

**DOI:** 10.1186/s12905-022-01768-0

**Published:** 2022-05-18

**Authors:** Gashaw Mehiret, Amsalu Molla, Aragaw Tesfaw

**Affiliations:** 1 Department of Medicine, College of Medicine and Health Science, Debre Tabor University, Debre Tabor, Ethiopia; 2 Department of Surgery, College of Medicine and Health Science, Debre Tabor University, Debre Tabor, Ethiopia; 3 Department of Public Health, College of Health Science, Debre Tabor University, Debre Tabor, Ethiopia

**Keywords:** Breast cancer, Knowledge, Practice, Debre Tabor University, Ethiopia

## Abstract

**Background:**

Breast cancer is the greatest common cancer in women worldwide, with approximately 1.7 million new cases diagnosed each year in the world which accounts for 12% of all new cancer cases and 25% of all cancers in women. Even though the higher mortality rate of breast cancer in low and middle-income countries, the practice of early detection methods is low and the majority of the patients who appeared at an advanced stage of the disease need palliative care with little survival rates. However, evidence is scarce on the knowledge and practice of breast cancer early detection methods among women of reproductive age in Ethiopia. Therefore we aimed to assess the knowledge on risk factors and practice of early detection methods of breast cancer among female graduating students of Debre Tabor University.

**Methods:**

Institution-based cross-sectional study was conducted. A stratified random sampling technique was used. Data were collected with a self-administer questionnaire. The collected data were processed and analyzed with the computer using SPSS version 25 software. Descriptive statistics were used to describe the socio-demographic information of participants. Binary and multivariable logistic regression with an adjusted odds ratio (AOR) and 95% confidence interval (CI) was used to identify factors associated with the outcome variable. Statistical significance was stated at p < 0.05.

**Result:**

A total of 270 female students participated in the study. The median age (± SD) was 24.63 years (± 1.26). All of the students heard about breast cancer and early detection methods. About 206 (76.67%) of the participants had good knowledge of breast self-examination. Mass media, health professionals, and friends in combination were the main source of information 172 (63.77%), only 110 (40.70%) of students performed a breast-self-examination and the rest did not perform it due to forgetting fullness and lack of knowledge. About 208 (77.1%) of the respondents respond family history is a risk factor for breast cancer. Being a health science student (AOR = 2.32; 95% CI: 2.12, 3.52), family history of breast problems (AOR = 3.41; 95% CI: 3.22, 8.33), and having a good level of knowledge (AOR = 1.83; 95% CI: 1.01, 5.68) were the factors associated with the practice of breast self-examination.

**Conclusion:**

Most of the participants were unaware of the benefits, appropriate timing, and techniques of doing a breast self-examination. Health science students had better awareness & practice of breast self-examination than non-health science students. Trained health professionals must give focus on giving health information regarding risk factors of breast cancer and methods of early detection of breast cancer to the public & their clients.

## Introduction

### Background information

Breast cancer is the most common cause of malignancy in women accounting for about one-third of all cancers in women is epithelial malignancies of the breast [1–3]. The Guideline of the American cancer society (2007) stated sixteen main known risk factors for breast cancer, among which gender is the most important risk factor as only less than one per cent of patients with breast cancer are males [4–6]. Lifestyle-related risk factors for breast cancer include having no children, oral contraceptive use, tobacco smoking, obesity, and alcohol use, no history of breastfeeding, and hormone replacement therapy. Breast cancer also occurs more commonly in women with a family history of breast cancer than in the general population [2, 7, 8].

Most breast cancers will present as a hard lump, which may be related to in drawing of the nipple. Although any portion of the breast, including the axillaries tale, may be involved, breast cancer is found most frequently in the upper outer quadrant of the breasts [2, 9, 10].

Carcinoma of the breast is one of the manageable cancers if noticed early. The American Cancer Society recommends as an option breast awareness & Breast self-examination (BSE) for early detection of breast cancer [7, 11, 12]. Breast cancer can be early detected with the combination of BSE, clinical breast examination by a caregiver, and mammography [2, 13].

Early detection of breast cancer cases before advancement is a crucial factor in the prognosis and survival of breast cancer patients [1]. Breast self-examination clinical breast examination and mammography are commonly used methods of early detection in the current era [8, 11]. Among these BSE is the recommended one particularly in developing countries meanwhile it was a simple, cost-free procedure and quick[12, 14].

As evidence displays, the incidence of breast cancer cases in Ethiopia is growing disturbingly and growing into the most common and most commonly diagnosed tumour among women with a projected incidence rate of 43 cases per 100,000 women [1]. According to the Addis Ababa population-based cancer registry report, breast cancer accounts for 33% of all female cancer cases and 23% of all cancers in the country [11, 15, 16].

If not diagnosed and treated early, breast cancer is life treating. Advanced stage and large tumour size at diagnosis are associated with decreased survival [2]. In Ethiopia, the majority of breast cancer patients are diagnosed at an advanced stage and their survival is poor as a result most patients need palliative care [8, 13].

Carcinoma of the breast occurs commonly in the western world, accounting for 3–5% of all deaths in women [4]. In resource-poor countries, it accounts for 1–3% of deaths [17]. Breast cancer is the most common cause of death in middle-aged women in western countries. In 2004, approximately one and a half million new cases were diagnosed worldwide [18, 19].

In low- and middle-income countries, it remains a significant public health challenge incidence rates have been shown to surge yearly by as much as 5% with over 1million projected new cases annually by 2020 [5].

Women with a family history of breast cancer are riskier than the general population, and yet this has far-reaching impacts in terms of counselling and tumour prevention in these women [20, 21]. Among the epithelial tumours in adults, breast cancer is unique in that effective screening improves survival. BSE, CBE by a caregiver, and mammography have been advocated as useful screening tools. Several trials have recommended that annual or biannual screening with mammography or mammography plus CBE in normal risk women over the age of 50 decreases breast cancer mortality [1, 22, 23]. Breast self-examination makes women more "breast aware", which in turn may lead to an earlier diagnosis of breast cancer. The rationale behind extending BSE practice as a screening test is the fact that breast cancer is frequently detected by women themselves without any other symptoms [24].

As evidence shows knowledge about breast cancer, family history of breast cancer, the presence of breast abnormalities, a higher year of study and is a health science student were the most important factors associated with the practice of breast cancer early detection methods [25–27].

## Methods and materials

### Study area and period

Debre Tabor is the biggest town in the south Gondar zone in the Amhara Regional State which is placed in the central highlands of Ethiopia and situated at a distance of 667Kms away from Addis Ababa in the Northeast direction. The geographical location of Debre Tabor town is 011″0.51 N.38.1″ E and 11.850′ N.38.017 E with an elevation of 2.706 m (8.878ft) above sea level. Debre Tabor University was established as a third-generation university in Ethiopia in 2011. Currently, the university had 6 main faculty. The study was conducted from October 1- to December 15, 2020.

### Study design

An institution-based cross-sectional study was conducted among DTU female graduating class students.

### Source population

The source population was all Debre Tabor university Female students.

### Study population

The study populations were all Debre Tabor university graduating female students.

### Study unit

The study unit was each female Debre Tabor university graduating student.

### Sampling technique and sample size determination

Sample size was computed based on single population proportion formula by using:

- 95% confident interval,
- 5% margin of error and.
- proportion based on research done on screening of breast cancer screening is
  $$\begin{aligned} {\text{n}} & = {\text{Z}}^{2} {\text{P}}\left( {1 - {\text{P}}} \right)/{\text{W}}^{2} \\ & = \frac{{\left( {{1}.{96}} \right)^{{2}} 0.{5}\left( {{1} - 0.{5}} \right)}}{{\left( {0.0{5}} \right)^{{2}} }} \\ & = 384 \\ \end{aligned}$$

Since our population was less than 10,000 we used correction formula (no = n/1 + n/N)

nf = 384/1 + 384/674 = 245 students

Then we added a 10% none response rate, the total sample size became 270.

P- Single population proportion for breast cancer screening to obtain the required sample size, from the total of 684 female graduating students stratified random sampling was applied to select study participants from the source population. First students were stratified by their department. Then, the sample size was proportionally allocated to all the departments in the institution based on the number of female students in each department, after which respondents were selected by simple random sampling technique. We took 40% representative from each department since the sample size is 270 which represent 40% of the total number of female graduating students.

### Data collection method and procedure

The data was collected by using a structured and self-administered questionnaire that was designed by reviewing previous similar studies [1, 11, 28, 29]. The questionnaire was pre-tested and evaluated by experts in the area to assess the face and content validity. The questionnaire was organized in English and it consists of all the variables that can come across the objectives of the study which are connected to socio-demographic characteristics, knowledge, and practice towards early detection methods of breast cancer and risk factors.

### Measurements

The study assessed the knowledge of risk factors and practice of participants towards risk factors and early detection methods of breast cancer. Students' knowledge regarding risk factors and BSE scores was calculated out of the total knowledge-specific questions. Each accurate response gets one point and zeroes for the incorrect one based on the respondent's response. Finally, the respondent who scores greater than or equal to the mean score were considered as having 'good knowledge and respondents who score less than the mean score was considered as having 'poor knowledge. There were 10 knowledge assessment questions and 8 practice assessment questions with a reliability coefficient of 76% and 78% respectively which was calculated using Cronbach’s alpha [1]. Before undertaking the data collection, the questionnaire was submitted to experts mainly oncologists, and other health professionals who are working in the area to test the face and content validity to assess whether they met the study objectives.

### Eligibility

#### Inclusion criteria

All-female graduating regular students of Debre Tabor University.

#### Exclusion criteria

Post basic and extension female graduating students were excluded from this study.

#### Variables

*Dependent variable* Practice of breast cancer early detection methods.

*Socio-demographic factors* age marital status, department, year of study.

*Personal and behavioral factors* alcohol, smoking, use of traditional treatment, lack of information about breast cancer, fear of exposing breasts fear of being diagnosed with cancer, and lack of trust in medical care.

*Clinical factors* previous history of benign breast disease, family history of breast cancer, previous history of any type of cancer, previous history of co-morbidities (hypertension DM, HIV), presenting symptoms.

### Operational definitions

Knowledge of breast self-examination: was measured by considering those who answered 70% and above of BSE questions as knowledgeable, and those who got < 70%non-knowledgeable [1].

*The practice of BSE* is defined as carrying out BSE at regular and the same time each month using a proper technique which is evidenced by the result of the questionnaire.

*Regular BSE* when BSE is done each month at the same time after some days of the menstrual cycle.

*Occasional BSE* when BSE is 1 to 3 times a year or every 3 months (irregularly at any time).

*Clinical breast self-examination (CBE)* when an experienced health worker examines breasts Statistical Analysis.

Mass media: Mass media refers to a diverse array of media technologies that reach a large audience via mass communication, e.g. Radio, Television, Facebook, telegram etc.

### Data quality management

To uphold data quality, ten data collectors and one supervisor who were health professionals (clinical nurses) were selected based on their experience in data collection and they were trained for 3 days. The questionnaire was developed by the research team based on questions used in the previous peer-reviewed published articles. The questionnaire was first prepared in English language and then translated to the local language (Amharic) to facilitate communication. The collected data was revised and checked for mistakes, legibility of handwriting, completeness, and consistency by the principal investigator and supervisor daily during data collection. Any mistake or ambiguity was cleared on the spot.

### Data processing and analysis

After data collection was finalized, the data were categorized, and then SPSS Version 25 program was used to enter and analyze knowledge and practice on early detection methods of breast cancer and other data. Suitable descriptive analysis (Median, SD) was used to describe the study population characteristics in terms of the variables and the results were presented by using tables and figures. The degrees of association between dependent and independent variables were assessed using OR and 95% CI. A P-value less than or equal to 0.05 was considered statistically significant. Bivariate and multivariate logistic regression analyses were conducted to identify the association of knowledge and practice of SBE with independent variables.

## Results

### Socio-demographic characteristics of study participant

A total of 270 regular female graduating students of DTU in the academic year of 2020/21 took part making the response rate 100%. The age of the participants ranged from 21 to 28 & the median age was 24.6 years (SD ± 1.26). Most of the participants were Amhara by ethnicity 109 (40.4%) and single regarding marital status 246 (91.1.1%). About 107 (39.63%) students were agricultural and other science students, 74 (27.4%) students were from technology, (4617.04%) were social science and humanity students, 25 (9.26%) were from health science students,18 (6.67%) were natural and computational science students (Table 1).

**Table 1:** Socio-demographic characteristics of study participants at Debre Tabor University, Northcentral Ethiopia, 2020/21

Variables		Frequency	Percentage (%)
Age (years)	20–25	203	75.2
	26–30	67	24.8
Ethnicity	Amhara	109	40.4
	Oromo	62	23.0
	Tigray	17	6.3
	SNPPRS	82	30.4
Marital status	Single	246	91.1
	Divorced	1	0.4
	Married	23	8.5
Department	Health science	25	9.26
	Technology	74	27.41
	Natural & computational science	18	6.67
	Agricultural & other science	107	39.63
	Social science & humanity	46	17.04

### Knowledge and practice on early detection methods of breast cancer.

All 270 participants (100%) heard about at least one of the three methods of early detection of breast cancer. Among this Only 55 (20.3%) students were aware of all the three methods (BSE, CBE & mammography), and 207 (76.7%) of respondents heard about breast self-examination as an early detection method of breast ca. more than half of 172 (63.8%) the students had gotten the information about early detection methods of breast cancer from mass media, health professionals, friends in combination and mass media was used as the only source of information in 65 (24%) of the students. 207 (76.7%) participants have good knowledge about BSE, but only 14 (6.8%) were aware of all the benefits of BSE. One hundred forty-five (53.6%) of them stated that early detection and treatment of breast Ca is the only benefit of BSE. Regarding knowledge of early detection methods among departments, 24 (96%) health science class students had good knowledge, and 183 (74.7%) of non-health science students had good knowledge about early detection methods of breast cancer.

### Awareness of breast self-examination of female graduating students:

Regarding awareness about breast self-examination from the total, the majority of the respondents 207 (76.67%) had good knowledge, and the rest of the respondents 63 (33.33%) had poor knowledge about Breast self-examination.

### Female students practice at the appropriate time for doing BSE

About 110 (40.74%) of the students performed breast self-examination within the past 12 months. The main reason for not performing BSE was forgetful fullness in 159 (58.8%) & lack of knowledge in 106 (39.4%) of the participants and the rest were due to fear of detecting abnormality and other issues. Of those who did BSE only 106 (39.3%) of the respondents did it regularly every month, 99 (36.7%) of them do BSE with the frequency of fewer than 3 times per year and about 63 (23.3%) of them did BSE only when they felt unhealthy. One hundred eighty-six (68.9%) of the students did SBE after menstruation and all of the respondents who performed SBE started doing it after 20 years of age. Breast self-examination techniques were known by 207 (76.7%) of the students and the wedge technique was used in 141 (52%) of the case, and the circular technique in 86 (32%).

Regarding Knowledge of things to be checked while doing BSE 76.7% of them knew at least two early signs of breast cancer. Among these 14 (6.8%) of them knew all the signs listed and the rest 193 (93.2%) of the students listed lumps in the breast, change in colour of the breast, nipple direction, and discharge as early signs of breast cancer (Table 2).

**Table 2:** Frequency distribution of female students practice on the appropriate time for doing Breast self-examination, at Debre Tabor University, Northcentral Ethiopia, 2020/21

Variables	Category	Frequency	Percentage (%)
Sources of information	Mass media	66	24.64
	Friends	8	2.90
	Health professional	24	8.70
	combined	172	63.77
Have you ever performed BSE during the past 12 months?	Yes	110	40.7
	No	160	59.3
How often do you perform BSE?	regularly each month	106	39.3
	< 3 times during the past 12 months	99	36.7
	> 3 times during the past 12 months	2	0.7
	Other	63	23.3
When do you perform SBE, concerning the menstrual period?	After menstruation	168	68.9
	Before menstruation	76	31.1
Which techniques do you use while performing BSE?	Vertical strip technique	2	0.8
	Circular technique	78	32.0
	Wedge technique	127	52.0
	Other	37	15.2

### Awareness of risk factors for breast cancer

Regarding the awareness of the risk factors for breast cancer, the most commonly known risk factor was a family history of breast cancer 208 (77.1%). Hormonal replacement therapy (HRT) use, lack of physical activity, high fat diet, ageing & late menopause were not identified as risk factors by most of the participants (Table 3).

**Table 3:** Frequency distribution of female student’s awareness of risk factors for breast cancer female graduating students, at Debre Tabor University, Northcentral Ethiopia, 2020/21

Risk factor	Knowledge of breast cancer					
	True		False		Don’t know	
	Frequency	%	Frequency	%	Frequency	%
Family history of breast cancer	208	77.1	3	1.2	59	21.7
Personal history of breast cancer	85	31.3	46	17.1	139	51.6
Early menarche	21	7.8	29	10.7	220	81.5
Late menopause	20	7.5	23	8.4	227	84.1
Aging	18	6.7	21	7.8	231	85.5
Alcohol	84	31	47	17.4	139	51.6
Late age at 1^st^ pregnancy	23	8.4	23	8.7	224	82.9
Never breast fed a child	85	31.3	46	17.1	139	51.6
Recent OCP use	52	19.4	25	9.3	193	71.3
Environmental pollution	85	31.3	46	17.1	139	51.6
High-fat diets	18	6.7	105	39.2	147	54.8
Tobacco smoke	85	31.3	46	17.1	139	51.6
Obesity (post-menopausal)	84	30.7	47	17.1	139	51.6
HRT use	83	30.7	18	6.7	169	62.6
Radiation to the chest	86	31.3	47	17.4	137	51.3
Lack of physical activity	15	5.5	107	39.8	148	54.8

### Factors associated with the practice of students on early detection methods of breast cancer

On bivariable logistic regression; age, knowing how to perform BSE, knowing when to perform BSE, and knowing how often to do BSE were positively associated with the practice Of BSE. Whereas, being a health science student and Family history of a breast problem and having a good level of knowledge remained significantly associated with the practice of BSE on the multivariable logistic regression. Participants who have good knowledge about breast cancer early detection methods were 1.83 times more likely to practice breast self-examination than those who have poor knowledge (AOR: 1.83; 95% CI: 1.01–5.68). The odds of practising BSE was 3.41 times higher among graduating female students who had a family history of breast problems than hadn't family history (AOR: 3.41 (96%CI; 3.8–33.16). Graduating students from health Science were 2.32times more likely to practice breast self-examination compared to no health graduating students (2.32, 95% CI: 2.12–3.52) (Table 4).

**Table 4:** Factors associated with the practice of breast self-examination among graduating students of Debre Tabor University, Northcentral Ethiopia, 2020/21

Characteristics		The practice of Breast Self-examination		COR with 95% CI	AOR with 95% CI	p-value
		Yes (n %)	No (n %)			
Being a health science student	Yes	19 (76)	6 (24)	4.75 (1.83,12.31)	2.32 (2.12, 3.52)	0.04
	No	147 (60)	98 (40)	1	1	
Family history of a breast problem	Yes	48 (69.5)	21 (30.5)	2.87 (1.60,5.16)	3.41 (3.22, 8.33)	0.001
	No	89 (44.3)	112 (55.7	1	1	
Having a good level of knowledge	Yes	64 (64.6)	35 (35.4)	3.86 (2.28,6.52)	1.83 (1.01, 5.68)	0.02
	No	55 (32.2)	116 (67.8	1	1	

## Discussion

Breast cancer is one of the most common causes of death & morbidity in middle-aged women but also is one of the manageable cancers if once detected early [17]. In this cross-sectional study 270, regular female students of Debre Tabor University with a median age of 21 years participated.

About 76.67% of the participants had good knowledge about breast self-examination which was lower than the figure from a study done in Saud Arabia which was 93.7% of the respondents were aware of BSE [21], But higher than the study figure from Debre Birhan university where (64%).such difference might be due to there was no health science students included on the research at Debre Birhan university or might be sample size difference[1, 10].

Mass media, health professionals, and friends were a detected source of the information in about 63.77% of the case and mass media was the only source of information in about 24.14% of them. There was some difference at Bahir Dar University where more than half (54%) of the students got the information from lectures (school) and mass media used in about only 22.1% of them [18]. The difference may be due to poor attitudes towards mass Media in some parts of the population. A similar study conducted at Debre Birhan University and the numbers on the source of information stated that about 40% of them got the information from mass media which was close to our study [5, 10, 29].

About 53.6% of the study participants knew the benefits of BSE though only 6.8% [14] were aware of all the benefits of BSE which is almost similar to the study at Ibadan was 53.2%[29].

Regarding breast self-examination practice, 40.7% of the respondents performed BSE within the past 12 months and 39.6% of them did BSE monthly, which is higher than at Debre Birhan University (28.3%) but lower than the study at Bahir Dar University which was 54.1% [10, 28]. A similar cross-sectional study was done in Nigeria [30], Austria [31] and Egypt [32, 33] on the practice of BSE, and the figure were 43.2%, 99%, and 10.4% respectively. Such difference might be explained by socio-demographic factors since local researchers had comparable figures. It might also be due to a lack of facilities for privacy and cultural misconception as doing repeated BSE increases the risk of acquiring cancer.

Regarding techniques of BSE from those who practised BSE 52% of the students practised wedge techniques and 32% of them used circular techniques. A similar study was done at Bahir Bar University and the figures were 22.1%, 12.6%, 9% for circular, wedge, and vertical strips respectively, and 41.1% used all of the three methods [1, 10].

Regarding Knowledge of things to be checked while doing BSE 76.7% of them knew at least two early signs of breast cancer. Among these 6.8%, [8] of them knew all the signs listed and the rest 93.2% (193) of the students' listed lumps in the breast, change in colour of the breast, nipple direction, and discharge as early signs of breast cancer. This figure is almost similar to the study at Bahir Dar University, lump in the breast was known by 90% of them, discharge from the breast by 85.8%, and discolouration or dimpling of the breast by 90% of them [1].

Regarding awareness of risk factors of breast cancer, the most commonly known risk factor was a family history of breast cancer (77.1%). HRT use, lack of physical activity, high fat diet, ageing & late menopause were not identified as risk factors by most of the participants. In contrast to the study at the Ibadan University of Nigeria where the personal history of breast Ca is the most known one [29]. A similar study was also done at Addis Ababa on nurses and family history of breast cancer 69.6%, smoking 54.4% were the most known risk factors which were comparable to our study [34]. There was also a similar study in Cameroon on risk factors awareness, radiation exposure (58.9%), hormone replacement therapy (58.2%), smoking (58.2%), alcohol consumption (46.7%), and high-fat diet (45.4%) were the most frequently indexed risk factors for breast cancer. Whereas late menopause (9.2%), early age at first menstruation (8.2%) and not having a child (7.9%) were the least recognized risk factors.

After controlling the effect of confounding factors good level of knowledge, having a history of a breast problem and being a health science student were the factors associated with the practice of breast cancer early detection. This finding is similar to other studies conducted in different countries [27, 35].

## Conclusion

Most of the students were unaware of the benefits, appropriate timing & techniques of doing breast self-examinations. Age of students, being a health science student, family history of a breast problem, and having a good level of knowledge were the factors associated with the practice of breast self-examination. Attention given to methods of early detection and risk factors for breast cancer by mass Media & health professionals was low.

Therefore based on the findings of our study, the following recommendations are forwarded;

Mass Media should give more focus on giving Health information regarding risk factors of breast cancer and practice of early detection methods for students.

Trained health professionals should give health education to their school friends, and families on breast cancer and breast self-examination.

Health education must be delivered to female students in high schools and higher institutions through gender clubs & organizations.

Further large-scale studies must be done in this area.

### Limitation of the study

Due to the cross-sectional nature of the study, we could not ascertain the cause-and-effect relationship between the factors and the outcome.

## Data Availability

The datasets used and/or analyzed during the current study are available from the corresponding author on reasonable request.

## Abbreviations

WHO: World Health Organization
DM: Diabetes Mellitus
HTN: Hypertension
CI: Confidence interval
COR: Crude odds ratio
AOR: Adjusted odds ratio
DTU: Debre Tabor University
